# Direct conversion of porcine primary fibroblasts into hepatocyte-like cells

**DOI:** 10.1038/s41598-021-88727-1

**Published:** 2021-04-29

**Authors:** Mariane Fráguas-Eggenschwiler, Reto Eggenschwiler, Jenny-Helena Söllner, Leon Cortnumme, Florian W. R. Vondran, Tobias Cantz, Michael Ott, Heiner Niemann

**Affiliations:** 1 Gastroenterology, Hepatology and Endocrinology Department, Hannover Medical School, Hannover, Germany; 2 Twincore Centre for Experimental and Clinical Infection Research, Hannover, Germany; 3 Translational Hepatology and Stem Cell Biology, REBIRTH - Research Center for Translational Regenerative Medicine and Department of Gastroenterology, Hepatology and Endocrinology, Hannover Medical School, Hannover, Germany; 4 Institute of Farm Animal Genetics, Friedrich-Loeffler-Institut (FLI), Mariensee, Neustadt, Germany; 5 Department of General, Visceral and Transplant Surgery, Hannover Medical School, Hannover, Germany; 6 German Centre for Infection Research (DZIF), Partner Site Hannover-Braunschweig, Tübingen, Germany

**Keywords:** Animal biotechnology, Genetic engineering, Differentiation, Reprogramming

## Abstract

The pig is an important model organism for biomedical research, mainly due to its extensive genetic, physiological and anatomical similarities with humans. Until date, direct conversion of somatic cells into hepatocyte-like cells (iHeps) has only been achieved in rodents and human cells. Here, we employed lentiviral vectors to screen a panel of 12 hepatic transcription factors (TF) for their potential to convert porcine fibroblasts into hepatocyte-like cells. We demonstrate for the first time, hepatic conversion of porcine somatic cells by over-expression of *CEBPα*, *FOXA1* and *HNF4α2* (3TF-piHeps)*.* Reprogrammed 3TF-piHeps display a hepatocyte-like morphology and show functional characteristics of hepatic cells, including albumin secretion, Dil-AcLDL uptake, storage of lipids and glycogen and activity of cytochrome P450 enzymes CYP1A2 and CYP2C33 (CYP2C9 in humans). Moreover, we show that markers of mature hepatocytes are highly expressed in 3TF-piHeps, while fibroblastic markers are reduced. We envision piHeps as useful cell sources for future studies on drug metabolism and toxicity as well as in vitro models for investigation of pig-to-human infectious diseases.

## Introduction

Pigs have a long standing and very successful history as biomedical model for studying human diseases and developing novel therapies, which is mainly attributed to the many genetic, anatomical and physiological similarities with humans^1–3^. This resemblance renders pigs important models for developing novel surgical techniques^4^, endoscopic approaches, such as NOTES (natural orifice transluminal endoscopic surgery)^5^ and even for complex metabolic disorders^6^. Additionally, pigs are a common food source, and, thus natural pathogens that cause infectious diseases with propensity to interspecies transmission such as endogenous retroviruses^7^, coronaviruses—CoVs^8^. Swine acute diarrhoea syndrome SADS-CoV^9^, and hepatitis E virus—HEV^10^, are a growing concern to human health.

For instance, pigs are asymptomatic natural reservoirs of HEV^11^. Chronic HEV infection is increasingly reported in immunosuppressed patients^12^, and can be highly lethal to pregnant women^13^. Recently, piglets were turned into animal models of chronic HEV by administrating immunosuppressive drugs^14^. However, while fecal HEV RNA levels have been detected in immunocompromised pigs until the end of the study, chronic HEV symptoms, such liver fibrosis or cirrhosis, which are commonly found in human patients, were absent. Thus, porcine hepatic in vitro models from easily accessible cell sources are desirable for future investigations of such diseases.

The availability of the porcine genome sequence and novel genome editing tools significantly expands the potential for producing new pig models for human diseases. Comparative genomic analysis has revealed high similarity between human and pigs^1,2^, including genes involved in xenobiotic metabolism such as cytochrome P450^15,16^. Porcine CYP450 orthologs are highly homologous to human enzymes^16,17^, rendering pigs as suitable models for drug studies. While there are numerous reasons for studying pig liver and porcine hepatocytes in vitro, the isolation and cultivation of primary hepatocytes is a challenging procedure^18,19^, often resulting in primary cells with low viability and short lifespan in the culture dish. Therefore, the in vitro generation of pig hepatocyte-like cells could provide a more stable and renewable cell source with on-demand availability for scientific and therapeutic studies on those important topics.

Hepatocyte-like cell (iHeps) generation via directed conversion of fibroblasts has been first described in mice, by transduction of mouse hepatic transcription factors (TFs) such as *Gata4*, *Foxa3* and *Hnf1α*^20^ or *Hnf4α* and different combinations of *Foxa1, Foxa2 and Foxa3*^21^. Both studies employed the *FOXA* family of transcription factors, known to be the major hepatic pioneer TFs, due to their ability of binding to nucleosomal DNA and opening the chromatin for further genetic modifications during liver development^22^. The generation of human iHeps (hiHeps) was described more recently^23,24^, and hiHeps were shown to be positive for albumin and α-fetoprotein (AFP), and displayed hepatic functions such as metabolism of drugs, urea production, glycogen and cholesterol storage^23,24^. Until date, the conversion of porcine somatic cells into pig iHeps (piHeps) has not yet been reported.

Here, we show for the first time the successful in vitro generation of piHep cells from primary adult fibroblasts via transduction of human transcription factors. To this end, we employed a three-phase screening of 12 different hepatic TFs for efficient generation of piHeps. We show that overexpression of a unique set of TFs, *CEBPα*, *FOXA1* and *HNF4α2* in porcine kidney fibroblasts (PKFs) results in cells with a morphology very similar to porcine primary hepatocytes (PPH) and several hallmarks of hepatic functions, such as low density protein uptake, Oil Red O and Periodic acid-Schiff (PAS) stainings indicating lipid and glycogen storage, respectively, thereby recapitulating features of porcine hepatocytes. Moreover, piHeps can metabolize drugs such as β-Naphthoflavone and Ibuprofen, thus demonstrating CYP450 enzyme activity, and express high levels of drug transporters, such as *SLCO2B1* and *ABCB1*. Together, we show as proof-of-principle the generation of directly converted piHeps. Such cells could be used in future studies of pig infectious diseases which have the potential to cross the species barriers to humans, such as endogenous retroviruses, coronaviruses and HEV infections, and as models for porcine drug discovery research.

## Results

### Experimental design and optimization for directed conversion of porcine fibroblasts towards a hepatic state

Twelve candidate hepatic transcription factors were selected based on previously published reports of successful hepatic directed differentiation from other mammalian species such as human and mice^20,21,23–25^. Thereafter, the degree of similarity between human and pig protein sequences was determined via sequence alignment for all 12 TFs using Geneious freeware^26^. All 12 TFs were highly conserved amongst human and pig species, ranging from 86 to 99.1% pairwise identity (Supplementary Table S1). All single TFs were cloned into lentiviral vectors for subsequent overexpression in PKFs.

**Figure 1:**
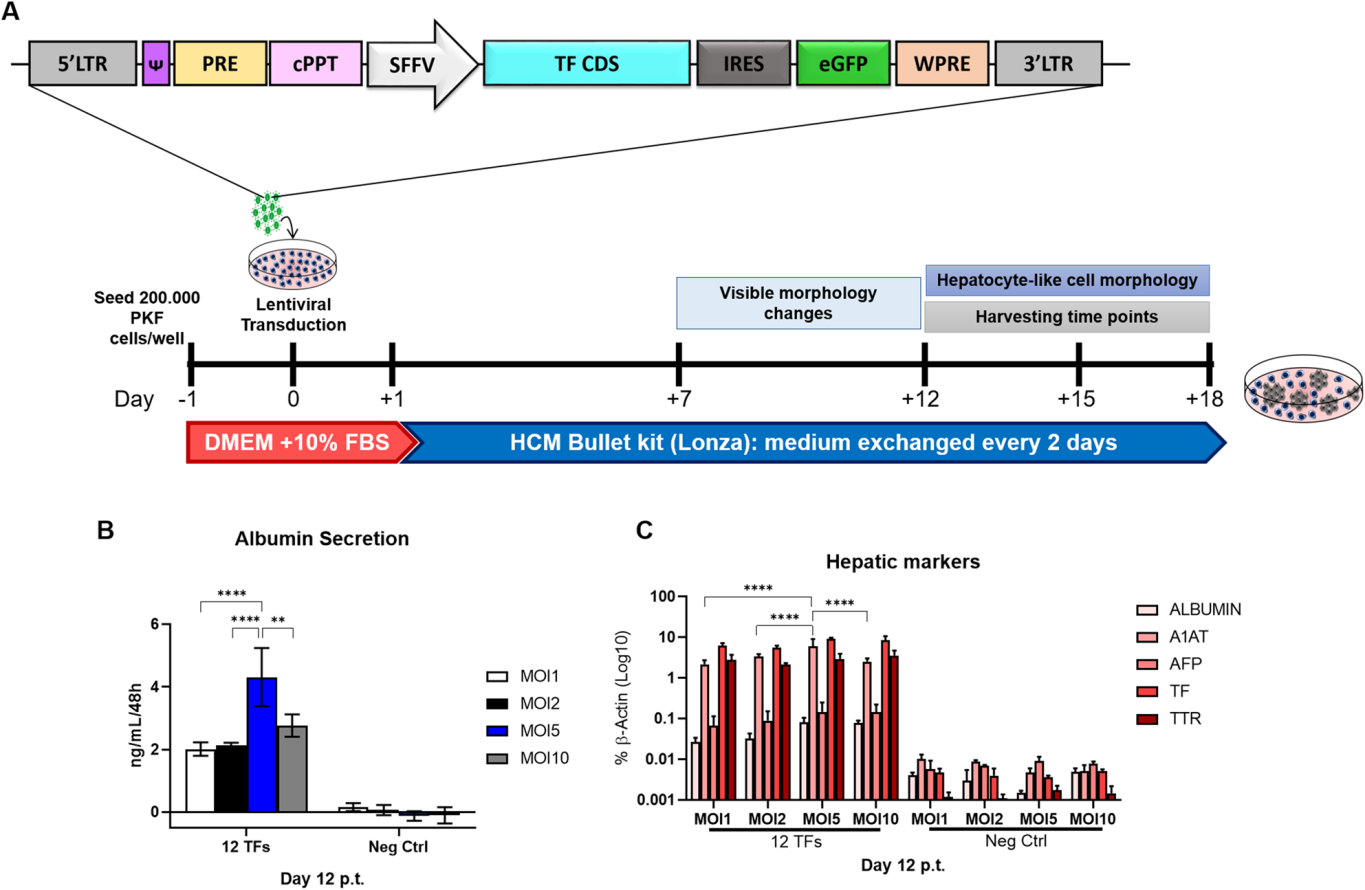
MOI determination for hepatic conversion of PKFs. (**A**) Schematic drawing of lentivirus vector design and cell culture protocol for directed hepatic conversion. (**B**) Albumin secretion quantification and (**C**) hepatic markers expression levels of cells directly reprogrammed with different MOIs per lentiviral vector of all 12 TFs, compared to Neg-Ctrl. All data were analyzed using two-way ANOVA, with Bonferroni’s post-test, except for *TF* gene, that was converted to mixed effects with Tukey’s multiple comparison post-test due to be missing one value. Significance from n = 3 independent values is displayed as *p < 0.05; ****p ≤ 0.0001.

In a first attempt, multiplicity of infection (MOIs) of 1, 2, 5 and 10 per vector were applied and cell supernatants were harvested at day 12 post-transduction (p.t.) for analysis of secreted porcine albumin by ELISA (Fig. 1A). Additionally, cells were harvested on the same day and used for total RNA extraction followed by qRT-PCR analysis of hepatic marker genes. PKFs transduced at MOI of 5 per TF-encoding lentiviral vector showed a significantly higher albumin secretion compared to other MOIs (Fig. 1B), whereas total gene expression levels of hepatic markers such as *ALBUMIN,* Alpha-1-antitrypsin—*A1AT;* Transthyretin—*TTR*, Transferrin—*TF* and Alpha-fetoprotein—*AFP*, were similarly induced in cells transduced with 12 TFs, regardless of MOI (Fig. 1C). Deciding that albumin secretion was the functionally most relevant parameter, a MOI of 5 per lentiviral vector was chosen for all following experiments. Subsequently, the time point for harvesting supernatants and transdifferentiated cells was further optimized. Thus, the experiments were extended up to days 15 and 18 p.t., denoted D15 and D18, respectively. In harvested supernatants, no significant difference of albumin secretion levels was found between D12, D15 and D18 (Supplementary Fig. S1a). However, qPCR-based expression analysis showed significant increases for *ALBUMIN*, *A1AT*, *TTR* and *TF* from D12 to D15, but no further increase was observed on D18 (Supplementary Fig. S1b). Therefore, the time point D15 was chosen for analysis of potentially directly reprogrammed hepatic-like cells. Taken together, a MOI of 5 per lentiviral vector and sample harvesting at D15 were chosen as most suitable experimental parameters for this direct hepatic reprogramming method and were used in all subsequent experiments.

### Three-phase transcription factor screening

**Figure 2:**
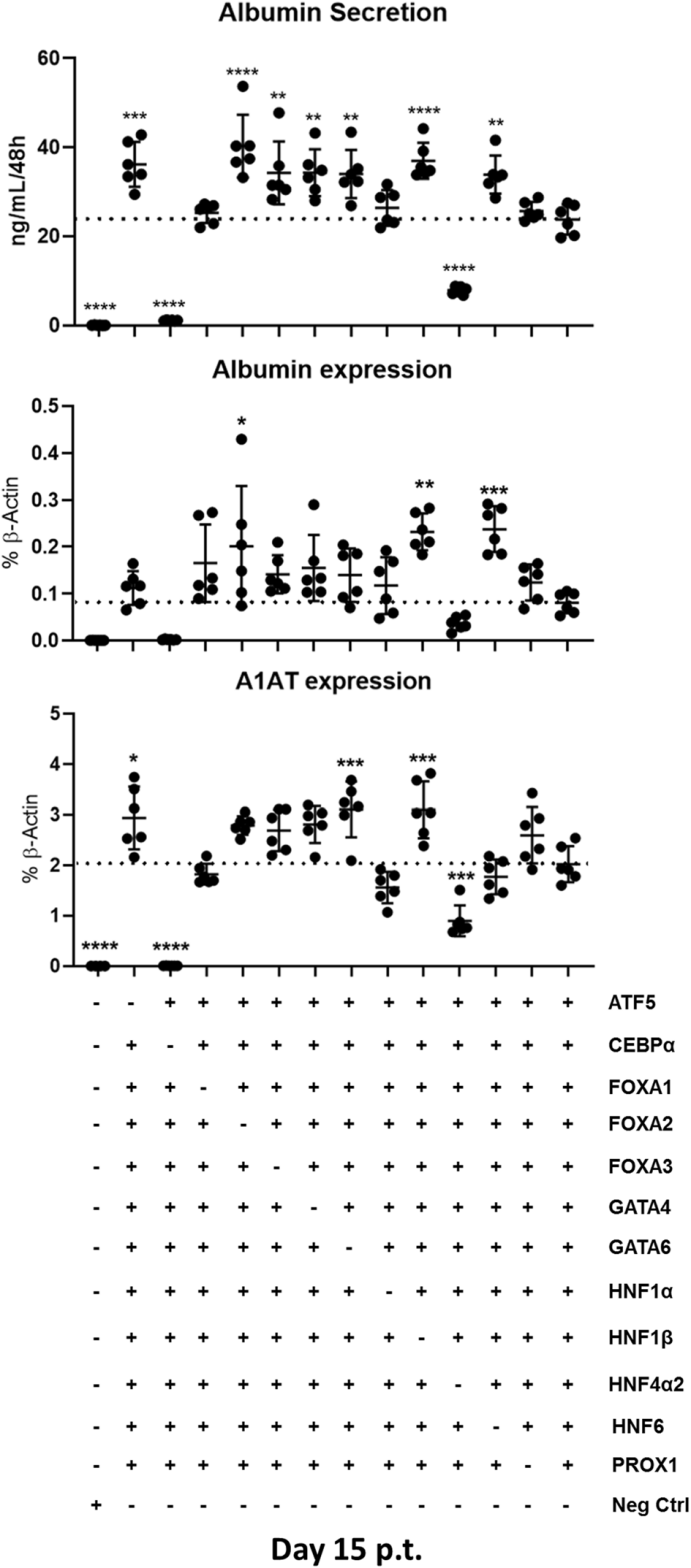
First step of transcription factor screening in PKFs. Albumin secretion (upper panel), *ALBUMIN* and *A1AT* gene expression levels (medium and lower panel, respectively) in PKFs transduced with 12 TFs minus one factor at a time (12-1 TF), compared to Neg-Ctrl. The dotted lines represent average values of 12 TFs replicates. All data were analyzed using one-way ANOVA, with Tukey’s post-test. Significance from n = 6 independent values is displayed as *p < 0.05; ****p ≤ 0.0001.

While a set of 12 TFs was clearly able to push porcine fibroblasts towards cells which display typical hepatic features not present in mock treated cells, it was possible that some of those TFs were more detrimental to the process than others, or that some factors were even impeding hepatic conversion. Therefore, a systematic screening of all 12 candidate TFs was performed to identify the most functional set of TFs for converting PKFs into hepatocyte-like cells. Initially, for the first phase of TF screening, a similar approach as the one described for iPSCs^27^ and mouse iHeps generation^20,21^ was employed, omitting one TF at a time, and using only the remaining TFs (’12-1’). In cells treated with all factors except for *CEBPα* or *HNF4α2,* low levels of albumin secretion and expression as well as low *A1AT* expression were observed (Fig. 2). Notably, omission of *ATF5*, *GATA4*, *GATA6* and *HNF1β* increased expression of hepatic markers, suggesting that they might be non-contributory to or possibly interfering with piHep generation. Surprisingly, omission of *FOXA1* and *PROX1* did not change expression of hepatic markers. Moreover, the omission of *FOXA1* did not reduce albumin secretion as well. However, the omission of either of the other two members of FOXA family, *FOXA2* and *FOXA3*, occasionally induced albumin secretion significantly higher than the 12 TFs, while *ALBUMIN* and *A1AT* expression levels remained at similar levels when compared to cells treated with all 12 TFs. Together, data from the separate omission of *FOXA1*, *2* or *3* suggested that an in-depth analysis is required to unravel the contributions of each of the factors to porcine hepatic reprogramming. Thus, in a second phase of TFs screening, the contribution of TF families, such as *FOXAs*, *GATAs, HNF1α* and HNF*1β*, was investigated by excluding the corresponding groups from the pool of 12 TFs. For the FOXA family all three *FOXAs* were omitted together, or two at a time (i.e.: -*FOXA1*/-*FOXA2* or -*FOXA1*/-*FOXA3* or -*FOXA2*/-*FOXA3*) while both *GATA* TFs or both *HNF1α* and *HNF1β* were simultaneously excluded from the pool of 12 TFs.

**Figure 3:**
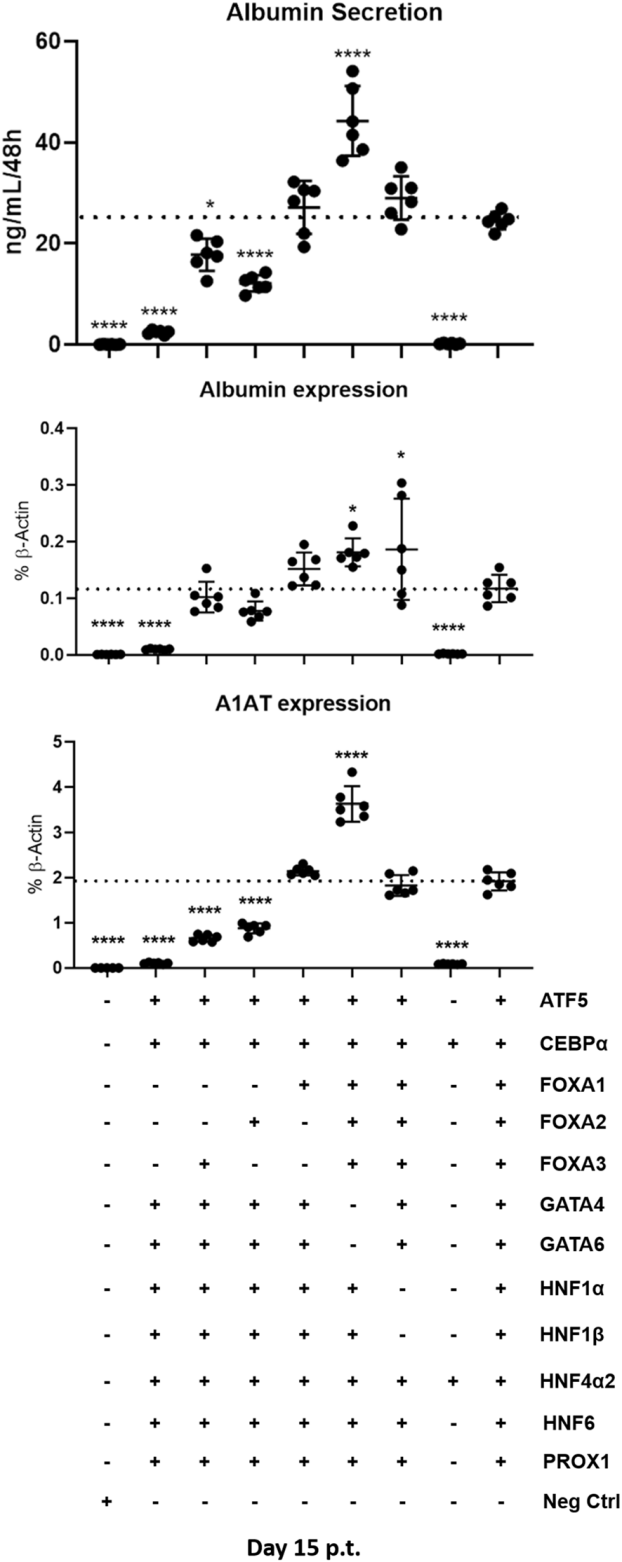
Second step of transcription factor screening in PKFs. Albumin secretion (upper panel), *ALBUMIN* and *A1AT* gene expression levels (medium and lower panel, respectively) in PKFs transduced with 12 TFs minus families of factors such as the *FOXAs*, *GATAs* and *HNF1α*; *HNF1β*. All data were analyzed using one-way ANOVA, with Tukey’s post-test. Significance from n = 6 independent values is displayed as *p < 0.05; ****p ≤ 0.0001.

Complete omission of all three *FOXAs* drastically impaired conversion of fibroblasts to piHeps while excluding only *FOXA2* and *FOXA3* had no effect on *ALBUMIN* and *A1AT* levels (Fig. 3). Furthermore, the omission of *FOXA1* in combinations with both other FOXAs (-*FOXA1*/-*FOXA2* or -*FOXA1*/-*FOXA3*) led to significantly decreased levels of hepatic markers, suggesting that *FOXA1* perhaps plays an important role in piHep generation (Fig. 3).

Furthermore, combined omission of *GATA4* and *GATA6* resulted in significantly increased albumin secretion and *A1AT* expression, suggesting that *GATA4* and *GATA6* are not critical for piHep conversion. Likewise, combined removal of *HNF1α* and *HNF1β* yielded significantly higher levels of albumin secretion and expression and *A1AT* expression, suggesting that these two factors were also not essential for directed hepatic conversion of PKFs.

Moreover, based on our findings from the first screen, we also tested whether transduction of PKFs only with *CEPBα* + *HNF4α2* (2TFs) could drive porcine hepatic reprogramming. Strikingly, 2TFs alone did not induce hallmark features of porcine hepatocyte-like cells, as low albumin secretion and *ALBUMIN* and *A1AT* expression levels were observed (Fig. 3). Altogether, the data from the second screening phase suggested that although *CEBPα* and *HNF4α2* were critically involved in the conversion of PKF cells to hepatocyte-like cells, other factors, most likely FOXAs, are also crucial for successful piHeps conversion.

**Figure 4:**
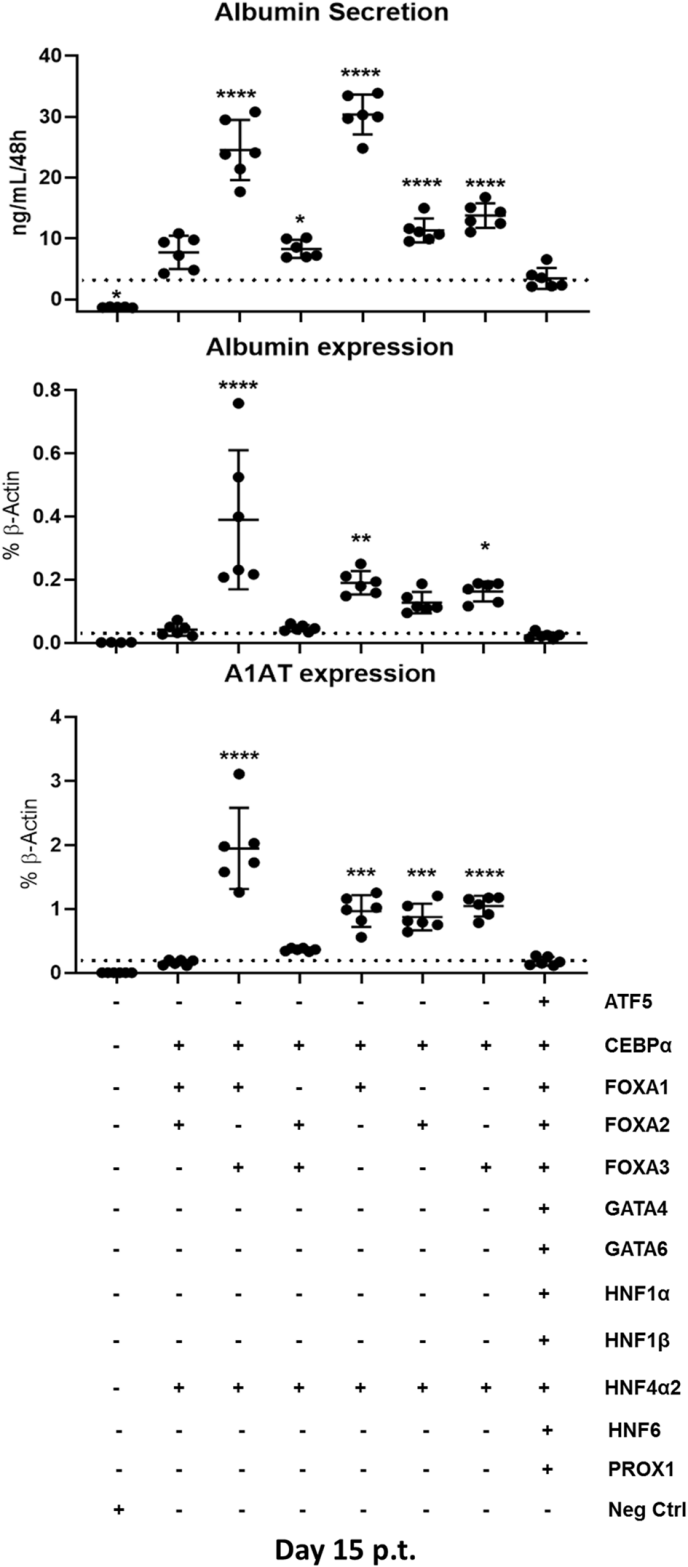
Third step of transcription factor screening in PKFs. Albumin secretion (upper panel), *ALBUMIN* and *A1AT* gene expression levels (medium and lower panel, respectively) in PKFs transduced with *CEBPα* and *HNF4α2* (2TFs) plus different combinations of *FOXAs* transcription factor family. All data were analyzed using one-way ANOVA, with Tukey’s post-test. Significance from n = 6 independent values is displayed as *p < 0.05; ****p ≤ 0.0001.

**Figure 5:**
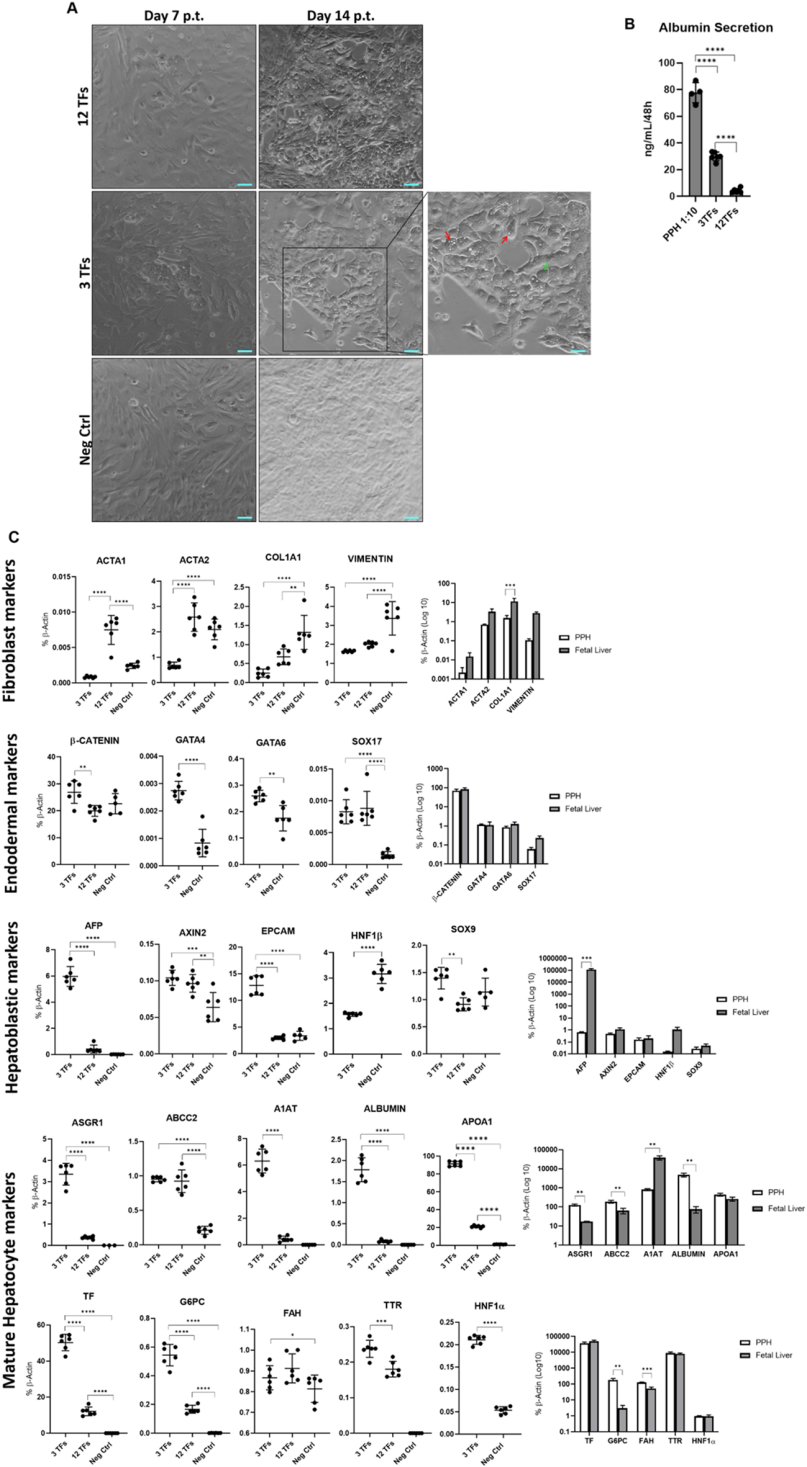
Morphology, Albumin secretion and gene expression analysis of 3TFs-piHeps. (**A**) Bright field pictures of PKF cells undergoing directed hepatic conversion at days 7 and 14 post-transduction (p.t.) with 3TFs *CEBPα*, *FOXA1* and *HNF4α2*, 12TFs or Neg-Ctrl. Blue scale bar equals to 100 µm. (**B**) Albumin secretion of 3TF-piHeps and 12TF-piHeps supernatants compared to PPHs supernatant diluted 1:10. (**C**) Expression of fibroblastic, endodermal, hepatoblastic and mature hepatic markers in PKF transduced with 3TFs: *CEBPα*, *FOXA1* and *HNF4α2*, 12TFs or Neg-Ctrl, as well as PPHs and Fetal liver samples. All data from qPCR of 3TF *vs* 12TFs *vs* Neg-Ctrl as well as Albumin ELISA were analyzed using one-way ANOVA, with Tukey’s post-test. qPCR data of PPHs *vs* Fetal liver samples were analyzed with multiple comparisons t-test. Significance from n = 3 independent values is displayed as *p < 0.05; ****p ≤ 0.0001. Genes *GATA4*, *GATA6*, *HNF1α*, *HNF1β* were analyzed only in 3TFs-piHeps and Neg-Ctrl, but not in 12TFs-piHeps, since the CDS of each lentiviral vector present in the 12TF pool would also be detected.

Therefore, the third phase of TF screening focused on investigation of reprogramming with the aid of 2TFs with different combinations of the *FOXA* factors. While addition of *FOXA1* + *FOXA2* or *FOXA2* + *FOXA3* to 2TFs did not affect expression of *ALBUMIN* and *A1AT* compared to 12 TFs, the addition of individual *FOXA*s (2TFs + *FOXA1*; 2TFs + *FOXA2* or 2TFs + *FOXA3*) led to increased levels of gene expression, where 2TFs + *FOXA1* showed higher albumin secretion levels than all other tested combinations. Interestingly, albumin secretion levels of 2TFs + *FOXA1* derived piHeps were higher than 2TFs + *FOXA1* + *FOXA3*, while the gene expression levels of *ALBUMIN* and *A1AT* were comparable in both samples (Fig. 4). Together, a combination of *FOXA1*, *CEBPα* and *HNF4α2* (from here on referred to as 3TFs) was determined as necessary and sufficient for conversion of porcine fibroblasts into piHeps. The 3TFs and 12TFs-piHeps readily exhibited visible morphological changes starting around D7 p.t., losing their initial fibroblastic elongated shape, turning into hexagonal cells with the emergence of small lipid vacuoles (red arrow), and more rarely, multinucleated cells (green arrow) (Fig. 5A). Noteworthy, albumin secretion was prominently higher in 3TFs-piHeps than in other conditions, reaching up to 33 ng/mL of albumin in an incubation period of 48 h, while freshly isolated primary porcine hepatocytes (PPHs) cultivated in collagen type-I and HCM medium secreted 770 ng/mL albumin in the same time period (Fig. 5B)*.*

### Gene expression of 3TF-piHeps

A profound switch in the gene expression signature, leading to activation of hepatic gene expression combined with lowered levels of fibroblastic markers is an important hallmark of successful iHep conversion^20,21,23–25^. Thus, expression analysis of fibroblast markers (*ACTA1*, *ACTA2*, *COL1A1* and *VIMENTIN*)*,* endodermal markers (*β-CATENIN, GATA4, GATA6, SOX17*)*,* hepatoblastic markers (*AFP, AXIN2, EPCAM, SOX9, HNF1β*)*,* and mature hepatocyte markers, including *TTR*, *TF*, *HNF1α*, *FAH* (Fumarylacetoacetate Hydrolase Enzyme), *G6PC* (Glucose 6-Phosphatase), *ASGR1* (Asialoglycoprotein Receptor 1), *APOA1* (Apolipoprotein A1) and *ABCC2* (ATP-Binding Cassette Sub-Family C member 2) were investigated in piHeps and Neg-Ctrl, as well as PPHs and fetal liver (FL) controls. Notably, fibroblastic genes showed significantly reduced expression levels (*ACTA1:* − 65%, *ACTA2:* − 33%, *COL1A1:* − 80%, *VIMENTIN:* − 49%) in 3TFs-piHeps compared to control; while *ACTA1* expression in 12TFs-piHeps was threefold higher than in the Neg-Ctrl (Fig. 5C). Interestingly, all endodermal markers were highly expressed in 3TFs-piHeps, and were expressed in FL and PPHs at similar levels. Next, in order to analyze whether 3TFs-piHeps represent a hepatoblastic stage, rather than mature hepatocytes, expression levels of hepatic progenitor markers *AFP, AXIN2, EPCAM, SOX9 and HNF1β* were compared between FL, PPHs and 3TFs-piHeps. *AFP* was higher expressed in FL than in 3TFs-piHeps, whereas *AFP* was more expressed in 3TFs-piHeps than PPHs (Fig. 5C). Furthermore, all remaining hepatoblastic/progenitor markers were higher expressed in 3TFs-piHeps compared to Neg-Ctrl, while *EPCAM*, *SOXA9* and *HNF1β* expression was increased in 3TF-piHeps compared to PPHs. *HNF1β* was twofold increased in Neg-Ctrl compared to 3TFs-piHeps, whereas for *HNF1α* the opposite was observed. Together, these data suggest that 3TFs-piHeps likely don’t have a complete mature hepatic phenotype (Fig. 5C). However, all analyzed mature hepatocyte markers were considerably higher expressed in 3TFs-piHeps compared to 12TFs-piHeps and Neg-Ctrl, including genes like *ASGR1*, *ALBUMIN* and *G6PC* that usually are preferentially expressed in adult hepatocytes.

Importantly, to fully resolve the genetic expression pattern of 3TFs-piHeps, more detailed gene expression characterization would be necessary. Taken together, although still at lower levels than PPHs, 3TFs-piHeps show increased expression of crucial hepatic markers compared to 12TFs-piHeps and Neg-Ctrl.

### Analysis of biological function of 3TF-piHeps

**Figure 6:**
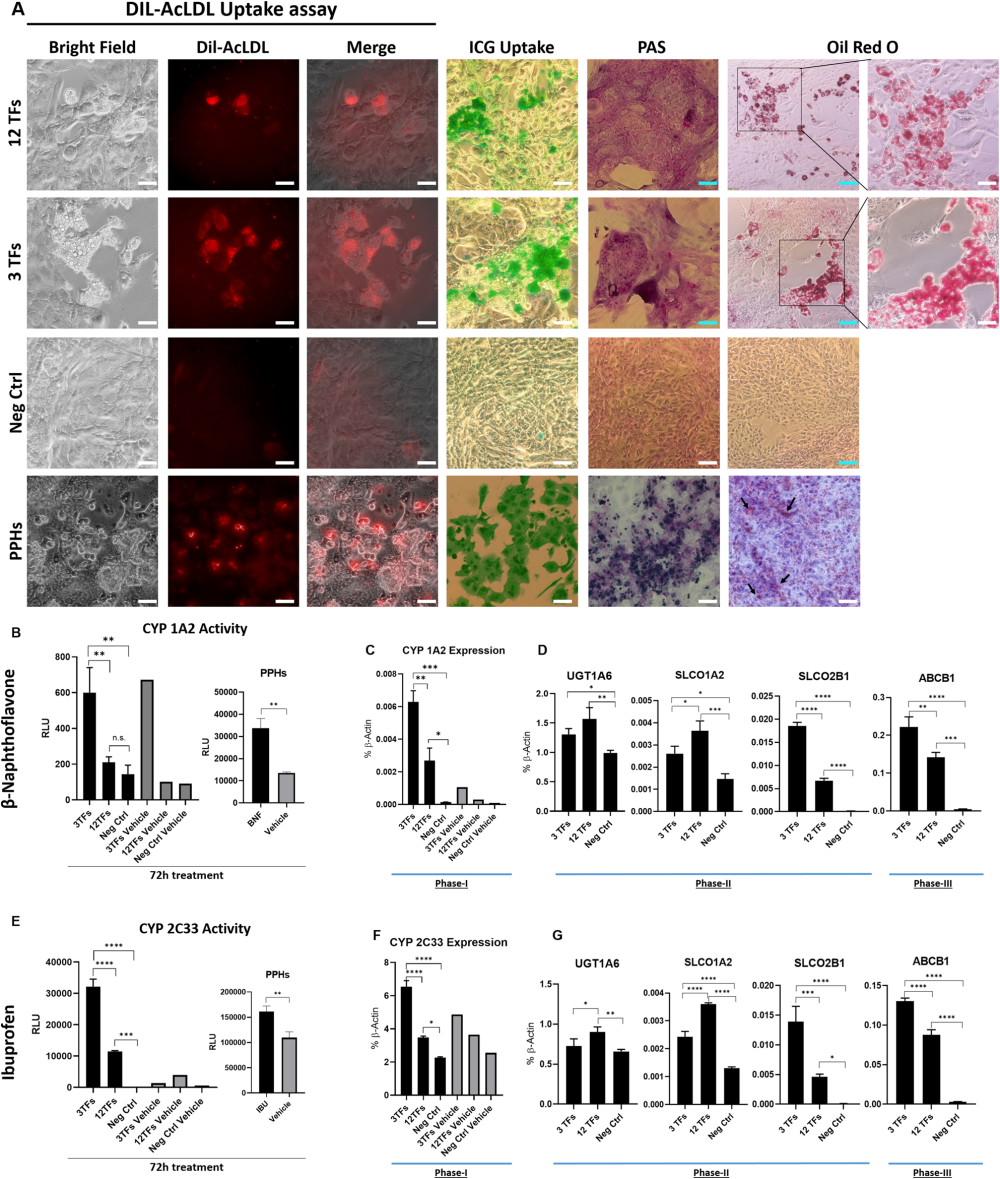
Functional analysis of 3TFs-piHeps. (**A**) From left to right: fluorescence pictures of Dil-AcLDL uptake assay (bright field, + 10 µg/mL conjugated Dil-AcLDL—Excitation 554 nm; and merge); bright field pictures of ICG uptake (1 mg/mL), PAS Staining’s and Oil Red O. White scale bars 50 µm and blue scale bars 100 µm. From top to bottom, cells transduced with 12TFs, 3TFs, Neg-Ctrl at day 15 p.t., and PPHs. (**B,E**) CYP450 assay normalized relative light unit (RLU) of luciferin showing CYP1A2 induction by 25 µM BNF and CYP2C9 (CYP2C33) induction by 500 µM IBU in piHeps, Neg-Ctrl and PPHs. (**C,F**) confirmation of *CYP1A2* and CYP2C33 gene expression levels by qRT-PCR. In pigs, the homologue gene for *CYP2C9* is *CYP2C33*. (**D,G**) Gene expression pattern of Phase-II and Phase-III of drug metabolizing genes *UGT1A6*, *SLCO1A2*, *SLCO2B1* and *ABCB1* were analyzed in 3TF-piHeps; 12TF-piHeps and Neg-Ctrl treated with BNF or IBU. All piHeps CYP450 data were obtained at day 16 post-transduction and analyzed using one-way ANOVA, with Tukey’s post-test, except for CYP induction in PPHs, where Unpaired *t*-test two-tailed was used. Significance from n = 3 independent values is displayed as *p < 0.05; ****p ≤ 0.0001.

Directly converted 3TFs-piHeps and 12TFs-piHeps were analyzed for hepatic functions such as glycogen storage, lipid accumulation, and CYP450 activity. Initially, the capacity of LDL protein uptake was analyzed. Cholesterol is usually transported in the blood stream by LDL lipoproteins, which are ultimately catabolized in the liver, releasing free cholesterol and amino acids^28^. A Dil-AcLDL uptake assay showed that 3TFs-piHeps and 12TFs-piHeps could take up LDL, similar to PPHs (Fig. 6A). Likewise, in a 4 h incubation period, 3TFs-piHeps and 12TFs-piHeps were capable of taking up the organic anion Indocyanine green—ICG, in a manner comparable to PPHs (Fig. 6A). Oil Red O staining revealed lipid storage in 3TFs-piHeps and 12TFs-piHeps as well as in PPHs (black arrows), which was absent in the Neg-Ctrl, and PAS staining for glycogen storage was also found in piHeps and in PPHs (Fig. 6A). Trace amounts of PAS-positive staining were also observed in the Neg-Ctrl, since PKFs can retain small amounts of glycogen as well^29^.

Another important functional characteristic of hepatocytes is the capability of metabolizing drug compounds, primarily via cytochrome 450 enzymes (CYP450), and by solute carrier transporters (SLCOs)^30^. In pigs, CYPs are found in various organs, but are predominantly active in liver and intestine^17^. By employing a protocol commonly used for measuring CYP450 activity in human cells, BNF and IBU were used for induction of CYP1A2 and CYP2C33 (the porcine ortholog to human CYP2C9), respectively, in 3TFs-piHeps, 12TFs-piHeps and Neg-Ctrl. The present data shows that both drugs, BNF and IBU, greatly induced CYP1A2 and CYP2C33 activity, respectively, in 3TFs-piHeps, compared to both 12TFs-piHeps and Neg-Ctrl cells (Fig. 6B,E). Induction of CYP1A2 and CYP2C33 was confirmed in PPHs (Fig. 6B,E). Furthermore, gene expression of *CYP1A2* and *CYP2C33* was confirmed in 3TFs-piHeps, 12TFs-piHeps and Neg-Ctrl, resulting in 45- and 2.9-fold increases in 3TFs-piHeps compared to Neg-Ctrl; while expression in 12TFs-piHeps was 19- and 1.53-fold increased when compared to Neg-Ctrl (Fig. 6C,F). Moreover, genes involved in the sub-sequential phases-II and -III of drug metabolism, such as *UGT1A6*, *SLCO2B1*, *SLCO1A2* and *ABCB1* were also analyzed. In cells treated with BNF, *UGT1A6* expression in 3TFs-piHeps was 1.32-fold higher than in Neg-Ctrl, while for cells treated with IBU an 1.11 fold increase was observed (Fig. 6D,G). Similarly, *SLCO1A2* expression was 1.79 and 1.86-fold higher in 3TFs-piHeps than in the Neg-Ctrl, for BNF and IBU treatments, respectively. Notably, *UGT1A6* and *SLCO1A2* were higher expressed in 12TFs-piHeps rather than 3TFs-piHeps, suggesting that possibly other TFs in the 12TFs panel were additionally inducing expression of those genes. Additionally, *SLCO2B1* was expressed 140 and 195-fold higher in 3TFs-piHeps than in the Neg-Ctrl, for BNF and IBU treatments, respectively; and *ABCB1* expression was 43 times higher in 3TFs-piHeps compared to Neg-Ctrl for both, BNF and IBU treatments. Lastly, both genes, *SLCO2B1* and *ABCB1*, were also induced in 12TFs-piHeps compared to Neg-Ctrl for both, BNF and IBU treatments. While for *SLCO2B1* 50- and 65-fold increases were observed for BNF and IBU, respectively, *ABCB1* gene showed 27- and 29-fold increases for the same treatments (Fig. 6D,G).

In summary, BNF and IBU were able to induce CYP1A2 and 2C33 activities in piHeps, respectively, albeit at higher levels in 3TFs-piHeps than in 12TFs-piHeps. Moreover, the activation of the subsequent phase-II and phase-III of drug metabolism was shown in 3TFs-piHeps cells, as well as for 12TFs-piHeps, demonstrating important functional aspects of piHeps.

Thus, 3TFs-piHep cells have acquired typical hepatic features such as inducing drug metabolism response (from phase-I to phase-III), glycogen storage and lipid droplet formation, as well as high expression of hepatic genes. These results provide compelling evidence as a proof-of-principle for successful directed conversion of porcine adult fibroblasts into functional hepatocyte-like cells.

## Discussion

Cellular reprogramming by over-expression of exogenous factors was first described in 1987, when mouse embryonic fibroblasts were converted into myoblasts by over-expression of *MyoD*^31^. Ever since, direct conversion of somatic cells into other somatic phenotypes by forced expression of selected lineage-specific TFs has been described in a variety of recent studies, ranging from direct differentiation of fibroblasts into cardiac cells^32^, neurons^33^, pancreatic cells^34^, and hepatocyte-like cells^20,21,23–25^.

Until date, in vitro generation of porcine hepatocyte-like cells had only been achieved with complex piPSC^35^ or pESC^36^ differentiation protocols. Noteworthy, piPSCs have been successfully generated by over-expression of human TFs^37^, suggesting that highly conserved human TFs can be successfully employed for cellular reprogramming of porcine cells. Therefore, we employed human hepatic TFs for porcine cellular reprogramming in the present study.

To date, direct conversion of pig somatic cells to another cell fate had been solely described for the generation of cardiomyocyte-like cells^38^, pancreatic β-cells^39^ and chondrocyte-like cells^40^, but not to hepatocyte-like cells. Our work shows for the first time a successful three phase screening procedure of human transcription factors for pig iHep generation.

Based on previously published protocols of iHeps generation in human and mouse, we chose 12 TFs that had been shown to be essential for iHeps generation^20,21,23–25^. We found high homology between protein sequences of human TFs and their porcine orthologues (Supplementary Table S1). In the first step, we adopted a strategy previously described for identification of reprogramming factors for iPSC generation^27^ and for miHep generation^20,21^, where one out of 12 TFs was omitted at a time (“12-1”). While the total number of iPSC colonies formed an exclusion parameter for TF candidates from the initial pool of 24 TFs^27^, for miHeps generation gene expression of hepatic markers was used as main criterion for defining if the respective TFs were indeed essential for piHeps formation^20,21^. We focused the screening on a combined analysis of functional albumin secretion and expression of mature hepatic genes *ALBUMIN* and *A1AT*. In a first stage, we observed clear evidence that *CEBPα* and *HNF4α2* were essential for piHeps reprogramming. *CEBPα* is a TF known to be highly abundant in the later hepatic maturation stage and adult liver^41^, activating hepatocyte-specific genes^42,43^, and contributing to iHeps generation and maturation^44^. Meanwhile, *HNF4α* is another key TF for hepatocyte development that has been extensively validated in successful iHep generation in mouse^21,25^ and human cells^23,24^, and in the present study we show that it plays a crucial role in piHeps generation as well. Currently, 12 transcript variants have been identified which can be expressed from the *HNF4α* locus. Thereof, *HNF4α2* is the longest variant resulting from alternative splicing of an *HNF4α1* transcript and comprising an additional 30 bp of coding sequence in the 3′ sequence^45^. Both *HNF4α1* and *HNF4α2* are enriched in hepatocytes and liver stem cells, while *HNF4α2* is homologous to the mouse *HNF4α*^46^. Both variants have been employed in previous studies for generation of human iHeps and are generally referred to as *HNF4a*^23,24,47^. However, an extended side-by-side comparison regarding specific consequences for hepatic transdifferentiation is still missing.

Through a second and third screening step, we were able to pinpoint that *GATA4*, *GATA6*, *HNF1α* and *HNF1β* TFs were dispensable for piHeps generation, whereas *FOXA* genes are important contributors to this process. Indeed, we found comparable levels of the TFs *GATA4* and *GATA6* in PPHs and FL, which were also induced in 3TF-piHeps. In the mouse, it was shown that both TFs are expressed in adult hepatocytes, and that depletion leads to minimal phenotypic changes in the adult liver^48^. Moreover, we found lower levels of *HNF1β* in PPHs compared to FL and observed reduced expression of this gene in 3TFs-piHeps compared to Neg-Ctrl. Thus, *HNF1β* is possibly not a suitable marker for characterizing the hepatoblast status in our context, since the fibroblasts of origin, PKFs, already express high levels of *HNF1β*. In contrast, *HNF1α* expression was elevated in 3TF-piHeps compared to controls. Together, our data corroborates previous findings showing that *HNF1β* is required during liver development^49,50^, while *HNF1α* is active in adult hepatocytes^49^. Importantly, we also show that 3TF-piHeps express several markers of functional adult hepatocytes, including the unique cell-surface marker *ASGR1*^51^.

Our results suggest that *FOXA1* is a crucial TF for piHeps generation and composes, together with *CEBPα* and *HNF4α2*, a ‘core’ group of 3 necessary TFs. *FOXA* and *GATA* TF families are known as pioneer TFs of hepatic lineage development, due to their ability of binding to nucleosomal DNA and consequentially opening the chromatin to further genetic modifications during liver development^22^. Interestingly, *FOXAs* were more effective in binding to nucleosomes than *GATAs*, but the underlying mechanisms remained unclear^22^. Moreover, it was recently shown in mouse cells undergoing iHep conversion by retroviral transduction of *HNF4α* + *FOXAs* that all *FOXAs* were able to bind to specific chromosomal regions and sequentially recruit HNF4α2 protein, thereby synergistically activating expression of hepatic genes^52^. A comparative analysis focusing on DNA binding sites of *CEBPα*, *HNF4α2* and *FOXA1* TFs in different mammalians, including humans, mice, rats, monkeys and dogs showed that deeply shared *cis*-regulatory modules—CRMs (stretches of the DNA where TFs binds to regulate gene transcription) were controlling genes involved in crucial liver regulatory functions^53^. Although pigs were not investigated in this study, it is possible that these CRMs are also similarly found in pigs. We suspect that *FOXA1* probably also acts as a pioneer TF in hepatic conversion of porcine somatic cells, synergistically cooperating with *HNF4α2* and *CEBPα* and actively promoting activation of hepatic-specific genes transcription during piHeps conversion. This assumption should be further investigated in future studies.

We demonstrate that our 3TFs-piHeps possess important features of hepatic morphology and perform hepatocyte-specific metabolic functions, such as ICG and Dil-AcLDL uptake, lipid and glycogen storage. Moreover, another important characteristic shown in reprogrammed hepatocytes is that they have CYP450 enzymatic activity^20,21,23–25,44^. In porcine liver, it was shown that CYP2A and CYP2D were the most abundant CYP enzymes, with relative protein quantities of 31% and 28% respectively; followed by CYP1A, CYP3A, CYP2C and CYP2E, with 4%, 14%, 10% and 13%, respectively^15,17^. CYP450 families share high homology in their nucleotide and amino acids composition between human and pigs: human CYP1A2, CYP2C9 and CYP3A4 shares 85%, 76% and 82% of amino acids homology with their porcine orthologs CYP1A2, CYP2C33 and CYP3A39, respectively^16^. This high CYP enzyme homology conveyed pigs as formidable models for drug metabolism studies^16^. Here, we show that PPHs greatly induced CYP activity, and that 3TFs-piHeps were also able to efficiently metabolize drugs via activation of CYP450 enzymes, such as CYP1A2 and CYP2C33, even more efficiently than 12TFs-piHeps. Additionally, we could also show that the subsequent phase-II and -III drug metabolism enzymes were also expressed in 3TFs-piHeps, indicating that 3TFs-piHeps could be good models for in vitro studies of drug toxicity and screening assays for new pharmaceuticals. Reprogrammed piHeps can be easily produced, and would provide a viable on-demand source of cells. We also envision potential employment of piHeps in the future for in vitro investigations of host–pathogen interaction mechanisms and cross-species transmission of infectious diseases such as SADS-CoV and HEV, where not always the live animal model satisfactorily mimics all symptoms found in clinical patients (e.g. for chronic HEV)^11,12^, or where high and fast lethality impairs extensive investigations (such as for SADS-CoV)^9^. Notably, swine infectious diseases are of concern to human health not only due to meat consumption; but also, because of pigs being potential organs donors for transplants (i.e. xenotransplantation), where existing pathogens could be fatal to immunosuppressed recipient patients.

Pigs are genetically similar to humans with a comparable biological anatomy and physiology, rendering them as an attractive host candidate for future generation of human organs. In this context, the in vivo generation of pig/pig chimeric organs has already been shown via blastocyst complementation (BC) of pancreatogenesis-disabled embryos with fluorescence marked blastomeres, resulting in chimeric foetuses with functional pancreata^54^. However, fluorescence marked donor cells were additionally found in all organs, including the brain. This ground-breaking work revealed a critical role of the cellular and genetic niche enabling organ formation in pigs, only preceded by studies in rodents^55^. Thereafter, human/pigs BC chimerism was similarly attempted. However, PSCs were increasingly being eliminated from post-implantation porcine blastocysts^56^, showing that interspecies barriers for this procedure might exist. Recently, it was shown that porcine mCherry^+^ pluripotent cells with expanded potential (termed pEPSC-mCherry^+^) contributed to both trophoblast and inner cell mass of porcine blastocysts; being traced also in several organs and placenta of chimeras derived from pEPSC-mCherry^+^ conceptuses, showing their potential to contribute neuronal tissue as well^57^. Moreover, human/pig BC is hampered by unresolved ethical issues, such as a possible contribution of human cells to the porcine neural system. Such neuronal contribution was recently shown in monkey/pig BC chimerism by analysis of primate mitochondrial integration in different tissues^58^. To overcome such issues, injection of differentiated cells in organ development impaired animal models would be an attractive approach.

The genetic knockout of fumarylacetoacetate hydrolase enzyme (FAH^−/−^) leads to hepatic development impairment in early porcine embryos/fetuses that can be treated with NTBC (2-(2-nitro-trifluoromethylbenzoyl)-1,3cyclohexanedione) to complete gestation^59^. Thus, pig/pig chimeric liver generation, without cellular contribution to other organs, could theoretically be achieved by application of reprogrammed iHeps, such as piHeps, into early FAH^-/-^ pig fetuses. Such pig/pig studies will greatly contribute to better comprehend chimeric mechanisms and help to advance relevant techniques aiming at a better understanding on how to overcome interspecies barriers for future human organ generation in pigs.

In conclusion, we show for the first time conversion of adult porcine fibroblasts directly into hepatic cells in vitro. We identified *CEBPα*, *FOXA1* and *HNF4α2* as essential transcription factors to induce piHeps which are functionally similar to primary hepatocytes and express important hepatic marker genes. PiHep cells could be an on-demand source of hepatic cells for in vitro studies of metabolic liver diseases, drug discovery and toxicity as well as molecular and genetic studies of infectious diseases with concern to human health. Ultimately, we envision piHeps as a potential source of cells for chimeric liver generation in pigs, paving the path for future interspecies chimerism experiments.

## Material and methods

### Plasmid construction and lentiviral vectors production

Plasmids harbouring coding sequences (CDS) of 12 human TFs were purchased from Addgene or Genscript (Supplementary Table S2). Plasmid pLX302_FOXA1-V5 was a gift from William Hahn (Addgene #70090); plasmid pSLIK 3XFLAG-wtGATA6-3XAU1 neo was a gift from Kevin Janes (Addgene #72618)^60^; and plasmids FR_HNF1β (Addgene #31101), FR_HNF4α2 (Addgene #31100) and FR_HNF6 (Addgene #31099) were a gift from Gerhart Ryffel^61^. For cloning of all 12 TFs into an eGFP expressing lentiviral vector construct, their respective CDS were PCR amplified and digested (Supplementary Table S2). Each CDS was then cloned into the lentiviral expression vector pRRL.PPT.SF.mOct34.i2GFPpre (gift from Axel Schambach, MHH^62^) in between a constitutive Spleen Focus Forming Virus (SFFV) promoter and an internal ribosome entry site element (IRES), followed by an eGFP (enhanced green fluorescent protein) CDS (Supplementary Fig. S1a), thereby replacing the mOct3/4 CDS with each respective TF’s CDS. The lentiviral vector pRRL.PPT.SF.GFPpre served as vector control (Neg-Ctrl) and was a gift from Axel Schambach, MHH^63^. All constructs were Sanger sequenced and sub-sequentially used for lentiviral production and titration in HEK-293T cells as previously described^64,65^. All primers used for cloning and sequencing are given in Supplementary Table S2. MOI was determined for directed conversion (Fig. 1b,c), where MOIs of 1, 2, 5 and 10 per lentivirus of the 12 TFs were used. Consequentially, for cells transduced with the negative control (Neg-Ctrl) MOI calculation was in accordance to the respective total lentivirus amount in each variable, resulting in total Neg-Ctrl MOIs of 12, 24, 60 and 120. For all other experiments, MOI of 5 per lentiviral vector was applied (total Neg-Ctrl MOI of 60).

### Primary porcine kidney fibroblasts (PKFs) isolation and culture

PKFs were isolated according to previously published protocol^66^, from porcine kidneys obtained from the slaughter house in FLI. Isolated cells were cultured in 0.1% gelatin-coated dishes in high-glucose DMEM with 10% fetal bovine serum (Sigma-Aldrich), 1% Penicillin/Streptomycin, 1% Sodium Pyruvate, 1% non-essential amino acids and 0.1% 2-mercaptoethanol. Cells were passaged using a 0.25% Trypsin–EDTA solution. All products were purchased from Thermo Fisher Scientific. Direct hepatic conversion experiments were performed with PKF cells at passage 4 previously tested for mycoplasma, in a 37 °C humidified incubator with 5% CO_2_.

### Primary porcine hepatocyte (PPH) isolation and culture

PPHs, used as positive controls for gene expression analysis and functional assays, were isolated and/or cultured according to previously published protocols^18,67,68^. Briefly, PPH were isolated from liver tissue obtained from landrace pigs undergoing full hepatectomy after euthanasia, using a 2-step collagenase perfusion technique. Single liver lobes were cannulated and flushed with pre-warmed (37 °C) 2.5 mM EGTA washing buffer. Thereafter, recirculating perfusion with a pre-warmed (37 °C) digestion buffer containing 0.05% collagenase P (Roche) was initiated. Upon sufficient digestion, the tissue was mechanically disrupted and the emerging cell suspension poured through a gauze-lined funnel followed by centrifugation (50×*g*, 5 min, 4 °C). The resulting cell pellet was washed with ice-cold PBS (50×*g*, 5 min, 4 °C), and cultured in Hepatocyte Maintenance Medium (HCM Bullet Kit, Lonza). PPH were plated at densities ranging from 1 × 10^6^ cells/well of a 6-well plate to 1 × 10^6^ cells/well in 12-well plates.

### In vitro directed hepatic conversion

PKFs were plated at a density of 200.000 cells/well in a 6-well plate coated with 0.1% gelatin with DMEM 10% FBS medium, the evening before lentiviral transduction. At the day of transduction, the medium was replaced with fresh DMEM 10% FBS + protamine sulfate (8 µg/mL) combined with the corresponding cocktail of lentiviral vectors (e.g., 10^6^ viral particles/vector/well for a MOI of 5). 24 h p.t., cells where washed with PBS 1× solution and the medium was substituted with HCM. The medium was replaced every 2 days until harvesting.

### RNA extraction, cDNA synthesis and quantitative RT-PCR (qRT-PCR)

Cells were lysed with RNA Lysis Buffer (PeqGOLD Total RNA kit, PeqLab), or TRIzol LS (Thermo Fisher Scientific), and kept at − 80 °C until total mRNA extraction, according to the suppliers instructions. Fetal liver (FL) pieces from wildtype slaughtered pig fetuses at day 25 of gestation, provided by FLI/Mariensee, were immediately flash frozen in liquid nitrogen and kept at − 80 °C until processing. FL pieces were first homogenized with TRIzol LS solution using stainless steel beads and processed by a TissueLyser LT device (Qiagen). The resultant homogenized solution was used for total mRNA extraction according to the TRIzol LS instructions protocol. Total mRNA from all samples was sub-sequentially treated with TURBO DNA-*free* Kit (Thermo Fisher Scientific), for complete removal of genomic DNA. The final product was quantified using the spectrophotometer Nanodrop N-1000 and processed for single stranded cDNA synthesis from 1 ug total mRNA in a final volume reaction of 10 uL using the High Capacity cDNA synthesis Kit (Thermo Fisher Scientific). The final cDNA was diluted 1:10 with RNAse and DNAse-free H_2_O and used for qRT-PCR using the StepOnePlus device (AppliedBiosystems). All TaqMan probes used in this study are listed in Supplementary Table S3.

### Enzyme-linked immunosorbent assay (ELISA)

Cell culture supernatants from reprogrammed cells, negative controls and PPHs were centrifuged at 17,000×*g* at + 4 °C for 20 min, and stored at − 80 °C until analysis. The PPH supernatant from confluent plates was concentrated in proteins, which required a 1:10 dilution in order to be able to fit this sample within the same standard curve with piHeps samples. The Porcine Albumin Quantitation Set (Bethyl Laboratories) was used for measuring Albumin secretion, according to the manufacturer’s instructions with minor modifications. Briefly, ELISA plates were coated with the Goat Anti-Pig Albumin Affinity Antibody (#A100-110A) diluted 1:100 in coating buffer (Carbonate-Bicarbonate, Sigma-Aldrich) and kept at + 4 °C overnight. In between each incubation step, plates were washed 5× with washing buffer (50 mM Tris, 0.14 M NaCl, 0.05% Tween20, pH 8.0). The next day, plates were blocked for 1 h at room temperature (R.T.) with 300 µL of Pierce protein-free blocking buffer (Thermo Fisher Scientific), and sub-sequentially incubated with samples and dilutions of pig serum as reference sample for the standard curve with n = 3 technical replicates for 1 h at R.T.. The standard curve consisted of a twofold serial dilution spanning from 50 to 1.5625 ng/mL, plus blank. The protocol was optimized to measure at high sensitivity, wherefore the secondary antibody Goat Anti-Pig Albumin HRP-conjugated antibody (#A100-110P) was used at a dilution of 1:30.000, and the plates were incubated in the dark for 1 h at R.T. Finally, the plates were incubated with 100 µL TMB solution (Sigma-Aldrich). The reaction was stopped with 100 µL stop buffer (1:1 dilution of 98% H_2_SO_4_ and ddH2O, resulting in a H_2_SO_4_ 9 M solution), and absorbance at 450 nm wavelength was measured with the plate reader Infinite M200 (Tecan). The 4-parameters-logistics curve fit with an open-source automated Excel spreadsheet by Andreas Swart* was used for determining the standard curve values and calculating Albumin concentrations in all experimental samples.

*http://www.rheumatologie-neuss.net/index_files/ELISA%20AUTO%20CURVE%20FIT.xlsm.

### CYP450 induction assay

CYP450 assays were performed and analyzed according to the manufacturer’s instructions. Briefly, cells were treated with 25 µM β-Naphthoflavone (BNF) or 500 µM Ibuprofen (IBU) for 72 h. At the day of the analysis, cells were incubated for 1 h with Luciferin-1A2 and for 4 h with Luciferin-H for CYP2C9, at 37 °C, respectively. CYP1A2 was analysed using kit #V8422 and, CYP2C33 was analysed using the kit #V8792, for CYP2C9 (Promega). For internal control of CYP induction specificity, in all analyzed conditions we used equivalent volumes of the vehicle substances corresponding to each drug: for BNF DMSO was used and for IBU Absolut Ethanol was used; at final concentrations of 0.1% and 0.05% respectively. The supernatant was harvested and incubated for 20 min at R.T. with luciferin detection reagent 1:1 dilution in a white plate, and resultant luminescence was measured using Tristar2 LB942 Multimode Reader and Instrument Control and Evaluation software (Berthold Technologies). The cells were then harvested for RNA extraction and gene expression analysis. For PPHs, the Beckman Coulter PARADIGM Detection Platform machine and Multimode Analysis software from Molecular Devices for CYP induction analysis was used.

### Dil-AcLDL and indocyanine green (ICG) uptake assays

Dil-AcLDL (Thermo Fisher Scientific) and ICG (Sigma-Aldrich) were diluted in HCM at the final concentrations of 10 µg/mL and 1 mg/mL, respectively. Cells were incubated with Dil-AcLDL or ICG dilution for 4 h at 37 °C, 5% CO_2_, followed by fixation (with 4% PFA solution) for 20 min at R.T., and analysed using regular (ICG) or fluorescence (Dil-AcLDL) microscopy (Olympus IX71 Microscope with Cell Sens Dimension Olympus software).

### PAS, oil red O staining

Periodic Acid Schiff (PAS) and Oil Red O stainings (Sigma-Aldrich) were performed, according to the manufacturer’s instructions, in cells previously fixed with 4% PFA solution for 20 min at R.T. Successfully stained glycogen storage and lipids were visualized under the microscope.

### Statistical analysis

All data were analyzed with GraphPad Prism 8.0, where error bars represent the standard deviation from at least n = 3 independent experiments. All graphics with single parameters and three or more variables, such as Albumin secretion and gene expression investigation, were evaluated using one-way ANOVA with Tukey’s post-test at 95% CI, whereas graphics composing of multiple parameters with more than three variables, such as multiple gene expression analysis, were statistically calculated using two-way ANOVA with Bonferroni’s multiple comparisons post-test. Multiple unpaired *t-test* was used for the statistical analysis of gene expression amongst PPH *vs* FL, whereas unpaired *t*-test two-tailed was used for CYP induction assay in PPHs. *P < 0.05, **P < 0.01, ***P < 0.001 and ****P < 0.0001.

### Ethical permit or wavers

Not Applicable. Tissue fragments were obtained from the slaughter house from FLI Institute or previously euthanased animals from MHH.

## Supplementary Information

Supplementary Information.

## Data Availability

The datasets used and/or analysed during the current study are available from the corresponding authors on reasonable request.
