# Supplementary material for: Direct conversion of porcine primary fibroblasts into hepatocyte-like cells
Source: Sci Rep. 2021 Apr 29;11:9334. doi: 10.1038/s41598-021-88727-1 (PMC8085017; doi:10.1038/s41598-021-88727-1)
Supplement: Supplementary file 1 — Supplementary Information. [file 41598_2021_88727_MOESM1_ESM.docx]

**Title: Direct Conversion of Porcine Primary Fibroblasts into Hepatocyte-Like Cells**

**Authors: Mariane Fráguas-Eggenschwiler^1,2*^; Reto Eggenschwiler^1,3^, Jenny-Helena Söllner^4^, Leon Cortnumme^3^; Florian W. R. Vondran^5^; Tobias Cantz^1,3^; Michael Ott^1,2^; Heiner Niemann^1,2,4*^.**

**^1^ Gastroenterology, Hepatology and Endocrinology** Department, Hannover Medical School, Hannover, Germany.

^2^ Twincore Centre for Experimental and Clinical Infection Research, Hannover, Germany.

^3^ Translational Hepatology and Stem Cell Biology, REBIRTH - Research Center for Translational Regenerative Medicine and Department of Gastroenterology, Hepatology and Endocrinology, Hannover Medical School, Hannover, Germany

^4^ Institute of Farm Animal Genetics, Friedrich-Loeffler-Institut (FLI), Mariensee, Neustadt, Germany.

^5^ Department of General, Visceral and Transplant Surgery, Hannover Medical School, Hannover, Germany. German Centre for Infection Research (DZIF), partner site Hannover-Braunschweig.

**^*^ = corresponding authors:** Fraguas-Eggenschwiler.Mariane@mh-hannover.de **and** Niemann.Heiner@mh-hannover.de


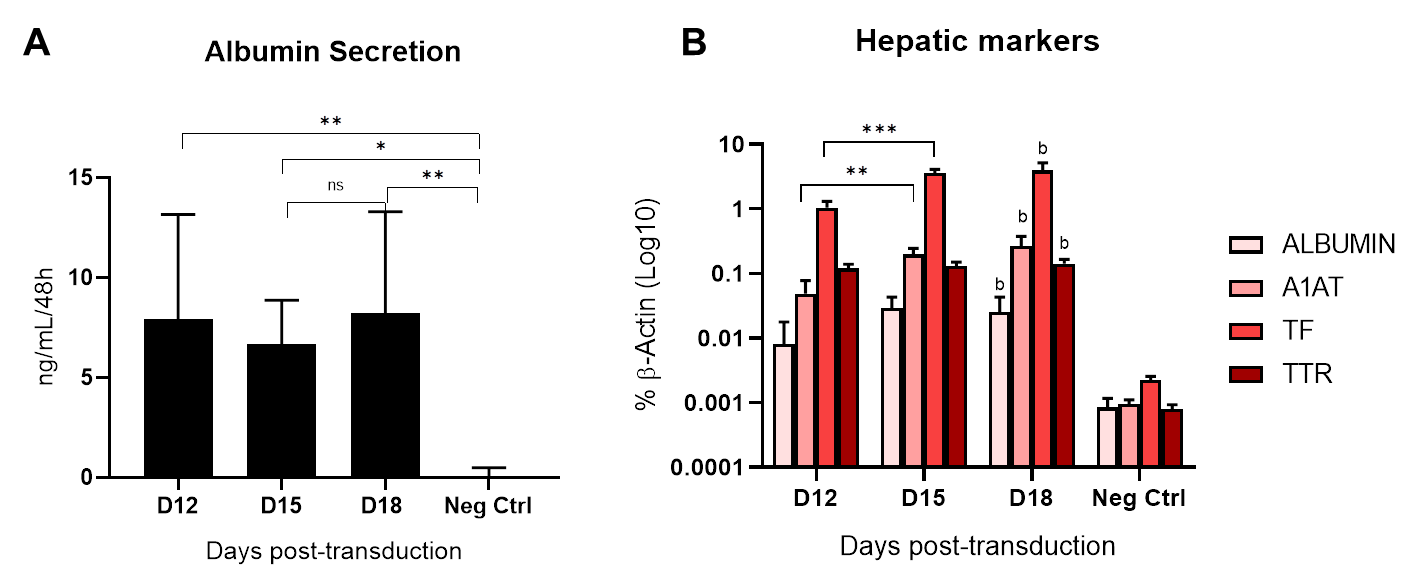


**Supplementary Figure 1: Determination of best time point of analysis.** (A) Albumin secretion quantification and (B) hepatic markers expression levels of cells direct converted with all 12TFs at MOI 5, compared to lentiviral backbone control (Neg Ctrl), harvested at different days post-transduction (D12, D15, D18). Albumin secretion data was analysed using one-way ANOVA, with Tukey’s post-test, while for the hepatic markers two-way ANOVA with Bonferroni’s post-test was used. Significance from n = 3 independent values is displayed as *: p < 0.05; **** p ≤ 0.0001, and “*b*” = statistically not significant for D15 *vs* D18 post-transduction.

**Supplementary Table 1: Similarities between protein sequences of human and porcine hepatic transcription factors.**

| **Transcription Factors** | ***Sus scrofa* protein sequence** | ***Homo sapiens* protein sequence** | **% pairwise identity of proteins (1)** |
| --- | --- | --- | --- |
| ***ATF5*** | XP_020950427.1  XP_013854133. | XP_011524931.1 | 86.0% |
| ***CEBPα*** | XP_003127063.1 | NP_001274353.1 | 95.5% |
| ***FOXA1*** | XP_001929311.1 | NP_004487.2 | 94.9% |
| ***FOXA2*** | XP_003134339.1  XP_005672811.1 | NP_710141.1 | 98.2% |
| ***FOXA3*** | XP_003127277.2 | NP_004488.2 | 92.0% |
| ***GATA4*** | XP_013845753.1 | NP_002043.2 | 94.1% |
| ***GATA6*** | NP_999493.2 | NP_005248.2 | 92.6% |
| ***HNF1α*** | NP_001027560.1 | NP_000536.6 | 97.1% |
| ***HNF1β*** | XP_020921865.1 | NP_001159395.1 | 97.7% |
| ***HNF6*** | XP_001929026.1 | NP_004489.1 | 99.1% |
| ***HNF4α2*** | XP_005673008.1 | NP_000448.3 | 97.7% |
| ***PROX1*** | NP_001121962.1 | NP_002754.2  NP_001257545.1 | 98.6% |

1. = % of identity of protein sequences between *Homo Sapiens* and *Sus Scrofa*

**Supplementary Table 2: Overview of molecular cloning strategies for generation of lentiviral vector plasmids.**

| **Transcription Factor** | **Plasmid ID**  **(serving as template for PCR)** | **Company / provider** | ***Homo sapiens* CDS** | **Primers** | **Buffer; DMSO; Annealing Temperature; extension time; cycles** |
| --- | --- | --- | --- | --- | --- |
| ***ATF5*** | OHu19993D | GenScript | XM_011526629.3 | ATF5-AgeI-for (AAAACCGGTATGTCACTCCTGGCGACC)  ATF5-NsiI-rev (AAAATGCATCTAGCAGCTACGGGTCCTCT) | Phusion GC buffer; 0%; Grad 55-65°C; 30s; 35x |
| ***CEBPα*** | OHu21531D | GenScript | NM_001287424.2 | CEBPα-AgeI-for (AAAACCGGTATGGAGTCGGCCGACTT)  CEBPα -NsiI-rev (AAAATGCATTCACGCGCAGTTGCC) | Kod Xtreme Hot Start; 0%; Grad 54-64°C, 60s, 35x |
| ***FOXA1*** | #70090 | Addgene | NM_004496.5 | FOXA1-AgeI-for (AAAACCGGTATGTTAGGAACTGTGAAGATGGAAG)  FOXA1-NsiI-rev (AAAATGCATCTAGGAAGTGTTTAGGACGGGTC) | Phusion HF Buffer;  5%; Grad 55-65°C, 60s, 35x |
| ***FOXA2*** | OHu31655D | GenScript | NM_153675.3 | FOXA2-AgeI-for (AAAACCGGTATGCTGGGAGCGGTG)  FOXA2-NsiI-rev (AAAATGCATTTAAGAGGAGTTCATAATGGGC) | Phusion HF Buffer;  5%; Grad 55-65°C, 60s, 35x |
| ***FOXA3*** | OHu25423D | GenScript | NM_004497.3 | FOXA3-AgeI-for (AAAACCGGTATGCTGGGCTCAGTGAAG)  FOXA3-NsiI+mut-rev (AATATGCATCTAGGACGCATTAAGCAAAGAGC) | Phusion HF Buffer;  5%; Grad 55-65°C, 60s, 35x |
| ***GATA4*** | OHu19154D | GenScript | NM_002052.5 | GATA4-AgeI-for (AAAACCGGTATGTATCAGAGCTTGGCCATG)  GATA4-NsiI-rev (AAAATGCATTTACGCAGTGATTATGTCCCC) | Phusion HF Buffer;  5%; Grad 55-65°C, 60s, 35x |
| ***GATA6*** | #72618 | Addgene | NM_005257.6 | GATA6-AgeI-for (AAAACCGGTATGGCCTTGACTGACGG)  GATA6-NsiI-rev (AAAATGCATTCAGGCCAGGGCCA) | Kod Xtreme Hot Start; 0%; Grad 54-64°C, 2ʼ30s, 35x |
| ***HNF1α*** | OHu25248D | GenScript | NM_000545.8 | HNF1α-AscI-SF-for (AAAGGCGCGCCAGTCCTCCGAATGGTTTCTAAACTGAGCCAGC)  HNF1α-BamHI-rev (AAAGGATCCTTACTGGGAGGAAGAGGCC) | Phusion HF Buffer;  5%; Grad 55-65°C, 60s, 35x |
| ***HNF1β*** | #31101 | Addgene | NM_001165923.4 | HNF1β- AscI-SF-for (AAAGGCGCGCCAGTCCTCCGAATGGTGTCCAAGCTCACGT)  HNF1β- BamHI-rev (AAAGGATCCTCACCAGGCTTGTAGAGGAC) | Phusion HF Buffer;  0%;64.9°C, 60s, 35x |
| ***HNF4α2*** | #31100 | Addgene | NM_000457.5 | HNF4a2-AgeI-for (AAAACCGGTATGCGACTCTCCAAAACCC)  HNF4a2-NsiI-rev (AAAATGCATCTAGATAACTTCCTGCTTGGTGATG) | Phusion HF Buffer;  0%;64.9°C, 60s, 35x |
| ***HNF6 (ONECUT)*** | #31099 | Addgene | NM_004498.4 | HNF6-AgeI-for (AAAACCGGTATGAACGCGCAGCTGAC)  HNF6-NsiI-rev (AAAATGCATTCATGCTTTGGTACAAGTGCT) | Phusion HF Buffer;  5%; Grad 55-65°C, 60s, 35x |
| ***PROX1*** | OHu10836D | GenScript | NM_002763.5 | PROX1-AscI-SF-for (TTTGGCGCGCCAGTCCTCCGAATGCCTGACCATGACAGC)  PROX1-BamHI-rev (AAAGGATCCCTACTCATGAAGCAGCTCTTGTAG) | Phusion HF Buffer;  5%;60.0°C, 60s, 35x |

**Supplementary Table 3: Pig (*Sus scrofa*) taqman probes.**

| **GENE SYMBOL** | **TAQMAN PROBE** |
| --- | --- |
| *Actin-β* | Ss03376563_uH |
| *A1AT* | Ss03394873_m1 |
| *ALBUMIN* | Ss03378640_u1 |
| *ACTA1* | Ss04245853_m1 |
| *COL1A1* | Ss03373340_m1 |
| *VIMENTIN* | Ss04330801_gH |
| *AFP* | Ss03384005_u1 |
| *TTR* | Ss03383377_u1 |
| *TF* | Ss03374732_m1 |
| *G6PC* | Ss03387281_u1 |
| *APOA1* | Ss03394891_m1 |
| *ABCC2* | Ss03373437_m1 |
| *FAH* | Ss06867081_m1 |
| *CYP1A2* | Ss04246171_m1 |
| *CYP2C33* | Ss03394922_m1 |
| *UGT1A6* | Ss06941854_m1 |
| *SLCO1A2* | Ss03375623_u1 |
| *SLCO2B1* | Ss06910571_m1 |
| *ABCB1* | Ss03373435_m1 |
| *HNF1β* | Ss03393022_u1 |
| *HNF1α* | Ss03392049_m1 |
| *GATA4* | Ss03383805_u1 |
| *GATA6* | Ss03384121_u1 |
| *ACTA2* | Ss04245588_m1 |
| *EPCAM* | Ss03384752_u1 |
| *SOX17* | Ss06862170_gH |
| *CTNNB1 (β-Catenin)* | Ss02667776_m1 |
| *AXIN2* | Ss06913912_g1 |
| *SOX9* | Ss03392406_m1 |
| *ASGR1* | Ss04327457_m1 |
